# Magnetic Superexchange and Mott Insulator Mechanisms in Cubic Perovskites: From First-Principles to Canonical Models

**DOI:** 10.1021/acs.inorgchem.5c01522

**Published:** 2025-06-24

**Authors:** Inés Sánchez-Movellán, Toraya Fernández-Ruiz, Richard Dronskowski, Ángel Martín-Pendás, Pablo García-Fernández, Miguel Moreno, José Antonio Aramburu

**Affiliations:** † Departamento CITIMAC, 16761 Universidad de Cantabria, Santander 39005, Spain; ‡ Institute of Inorganic Chemistry, RWTH Aachen University, Aachen 52074, Germany; § Departamento de Química Física y Analítica, 16763 Universidad de Oviedo, Oviedo 33006, Spain

## Abstract

The ground state of many insulating, open-shell transition-metal perovskites with a 180° metal–ligand–metal bridge is antiferromagnetic (AFM), as predicted by Anderson’s superexchange interaction or Hubbard’s model. These well-established, standard models show how these systems are insulators due to the minimization of the interactions between electrons, at the cost of localizing the electrons on the metal ions. In this work, we carry out first-principles simulations on the cubic perovskites KNiF_3_ and KVF_3_, analyzing electron densities, energies and bond indices. Although our calculations predict an antiferromagnetic ordering (AFM), in agreement with canonical superexchange models, we show through various indicators that the stabilization of this phase is not mainly associated with the antibonding magnetic orbitals but rather with bonding orbitals not included in the models. In particular, these traditional descriptions of superexchange do not adequately describe the ligand-to-metal electronic backdonation, which is an important element for stabilizing the insulating state of the two studied perovskite fluorides, albeit by diametrically different mechanisms: (1) reducing electron–electron repulsion in KNiF_3_, as proposed by Hubbard, whereas (2) enhancing electron–nuclear attraction in KVF_3_. Our findings highlight some of the limitations of these foundational models and offer a novel perspective on the understanding of magnetism.

## Graphical abstract

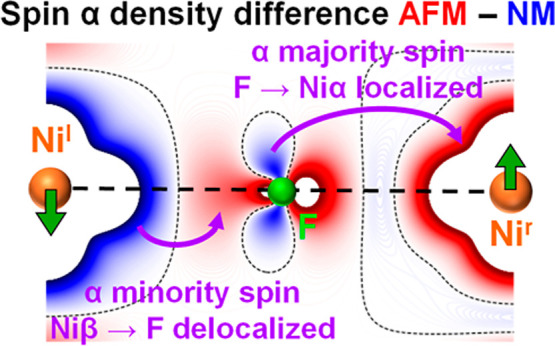

## Introduction

A fundamental challenge in the realm of insulating transition metal (TM) compounds is reaching a quantitative understanding of the origin of the tiny energy differences per magnetic ion (in the range 10–100 meV) between the ferromagnetic (FM) and antiferromagnetic (AFM) phases. Essential insights were provided by Anderson’s superexchange model^1,2^ or some of its variants, such as the one proposed by Hay, Thibeault and Hoffmann (H–T–H),^3^ but also by Hubbard’s model.^4^ Interestingly, the latter also underpins how these systems, which at first sight should be band metals, become insulators.^5^ The basic ingredients of these models are the same: (i) a one-electron Hamiltonian that describes the electron hopping between metal ions, and (ii) a strong electron–electron repulsion between electrons that are placed on the same ion. Typically (i) comes in the form of tight-binding with a minimal basis including only the magnetic orbitals (MOs), while (ii) is expressed through Hubbard’s parameter *U*. Despite their simplicity, these models have been successful for explaining, as a first approach, the nature of the magnetic ordering displayed by a huge range of materials with diverse composition and bonding types.^6,7^

In this work we explore, with the help of first-principles simulations, the cubic magnetic insulators KNiF_3_ and KVF_3_, whose magnetic orbitals are, respectively, σ (Ni^2+^, d^8^, t_2g_^6^e_g_^2^, *S* = 1) and π (V^2+^, d^3^, t_2g_^3^, *S* = 3/2). We find that, while these two archetypal systems are AFM, as predicted by the models above, the mechanisms leading to this result differ in each case and diverge from the unified interpretation usually offered by these theories. In particular, KNiF_3_ becomes an insulating magnetic material to reduce the electron–electron interaction, like in Hubbard’s model, while KVF_3_ does the same to increase the electron–nuclear attraction, an effect not considered in the model. In line with recent research on the origin of Hund’s rule^8–10^ or the magnetism of 3d metals,^11^ our main conclusion is that minimal-basis models, that only consider antibonding magnetic orbitals, cannot capture the fine details of the relaxation of the electronic structure. In particular, first-principles show that deep bonding orbitals are fundamental to understand the stabilization of the observed phase. In spite of the previous criticism, classical models are able to predict the correct state as they adequately consider the symmetry-breaking processes that allow electron localization. It is worth noting that Pascale et al.^12–14^ have recently published a series of interesting papers exploring the origin of superexchange in cubic perovskites using first-principles simulations of FM and AFM phases. We believe that the spin-density maps used in these works, however, have a scale that, as we will see later, is too coarse to discuss the subtle differences between both phases. Moreover, the interpretation of these maps relies on the qualitative concept of Pauli repulsion,^15^ that is not directly reflected in the interactions present in the Hamiltonian. In agreement with various results based on first-principles,^8–11^ we suggest that focusing on the analytical models described above provides a more precise approach to understanding the origin of superexchange.

## Computational Methods

All first-principles calculations have been performed in the framework of the spin-unrestricted Kohn–Sham density functional theory (DFT) with Crystal23 (localized orbitals)^16^ and VASP (plane waves)^17,18^ codes. Computational details of the calculations can be found in Section S1 of the Supporting Information. Both programs lead to comparable results and reproduce the lattice parameter of the stable AFM phase of KNiF_3_ and KVF_3_ within 1% of accuracy (see Table S1 in the Supporting Information). In both perovskites, we have first investigated the nonmagnetic (NM) phase, where α and β spin–orbitals are forced to have the same spatial distribution (spin-restricted calculation) leading to a fictitious metallic state (see Figures S2 and S3). Then, allowing for larger variational freedom by including spin polarization, the FM and AFM phases have been calculated, resulting, in both cases, in insulating states. The results have been analyzed using density difference maps between the different states and quantitative bond indices as the crystal orbital Hamilton population^19^ (COHP) and crystal orbital bond index^20^ (COBI), obtained with the LOBSTER^21^ suite.

## Results and Discussion

Although the present study is based on some of the ideas of the seminal paper by Landrum and Dronskowski^11^ (L–D) on the origin of the ferromagnetism in the elemental metals Fe, Co and Ni, it faces steeper difficulties, as differences in electron density, bonding and energies between the FM and AFM phases in cubic fluoroperovskites are more than an order of magnitude smaller than those of each individual phase with respect to the NM phase. These changes can be immediately appreciated in the energies and Mulliken populations calculated for the NM, FM and AFM phases displayed in Tables 1 and 2. As shown in Table 1, the energy difference between the metallic NM and the insulating phases is equal to 2.6 eV for KNiF_3_ and nearly 4 eV for KVF_3_ which is consistent with the insulating character of both perovskites. In contrast, the calculated energy difference, Δ*E*, between the FM and AFM phases of KNiF_3_ amounts to only 82 meV. Writing the effective exchange interaction as Σ*J*_*ij*_ *S*_*i*_ *S*_*j*_ and considering only the interaction among the six nearest cations, it leads to an exchange constant *J* = 9 meV, essentially coincident with the value derived by De Jongh and Block (8.5 ± 0.7 meV)^22^ from experimental measurements in pure KNiF_3_, as well as for nickel pairs^23,24^ formed in KMgF_3_:Ni^2+^. The calculated value for KVF_3_, Δ*E* = 34 meV, leads to *J* = 1.9 meV. This significant difference between the exchange constant of two perovskites already reflects that the unpaired electrons in KNiF_3_ exhibit σ-bonding, while in KVF_3_ there is a much weaker π-bonding, as shown in Table 2. It is also qualitatively consistent with the Néel temperatures of both systems, *T*_N_ = 246 K in KNiF_3_ ^22^ and *T*_N_ ≈ 50 K for KVF_3_.^25^

**Table 1:** Energy Differences between NM, FM and AFM States Broken Down by the Contributions to the Total Energy of KNiF_3_ and KVF_3_ ^a^

	KNiF_3_			KVF_3_		
energy diff	FM – NM	AFM – NM	FM – AFM	FM – NM	AFM – NM	FM – AFM
Δ*E*	–2.559	–2.640	+0.082	–3.886	–3.919	+0.034
Δ*V*_ee_	–19.655	–18.180	–1.475	+19.479	+18.895	+0.584
Δ*V*_en_	+11.520	+10.569	+0.951	–29.195	–28.332	–0.863
Δ*V*ee + Δ*V*en	–8.135	–7.611	–0.524	–9.716	–9.437	–0.279
Δ*T*	+7.310	+6.611	+0.699	+9.273	+8.887	+0.386
Δ*E*_XC_	–1.734	–1.640	–0.093	–3.443	–3.369	–0.073

a The contributions include electron-electron repulsion (*V*_ee_), electron-nuclei attraction (*V*_en_), kinetic (*T*), exchange-correlation (*E*_XC_) and total energy (*E*). All energies are given in eV per formula unit. Note that the Δ*V*_ee_ + Δ*V*_en_ contribution to the FM – AFM difference is around five times higher than that from the exchange-correlation, Δ*E*_XC_.

**Table 2:** Total Number of Electrons, *N*(e^–^), Including Core and Semicore Levels, for M^2+^ and F^–^ Ions in Cubic Perovskites KMF_3_ (M = Ni, V) for the NM, FM and AFM Phases (Using Mulliken Criterion; Values Derived from Löwdin and Bader Criteria are Consistent with These Results, See Table S3 in the Supporting Information)^a^

	KNiF_3_				KVF_3_			
	NM	FM	AFM	FM – AFM	NM	FM	AFM	FM – AFM
M^2+^ dσ *N*(e^–^)	2.520	2.288	2.310	–0.022	0.394	0.382	0.382	0.000
M^2+^ dπ *N*(e^–^)	6.000	6.009	6.009	0.000	3.087	3.033	3.042	–0.009
M^2+^ total *N*(e^–^)	26.880	26.687	26.704	–0.017	21.860	21.761	21.769	–0.008
M^2+^ charge	+1.12	+1.313	+1.296	+0.017	+1.14	+1.239	+1.231	+0.008
F^–^ pσ *N*(e^–^)	1.752	1.825	1.819	+0.006	1.831	1.842	1.841	+0.001
F^–^ pπ *N*(e^–^)	3.974	3.968	3.970	–0.002	3.918	3.944	3.940	+0.004
F^–^ total *N*(e^–^)	9.656	9.717	9.714	+0.003	9.656	9.694	9.689	+0.005
F^–^ charge	–0.656	–0.717	–0.714	–0.003	–0.656	–0.694	–0.689	–0.005

a The contributions to the total number of electrons for each orbital (d in M^2+^ and p in F^–^) are divided into σ and π contributions. The net charge of M^2+^ and F^–^ ions is also collected.

As in the L–D work on metallic Fe, our starting point is the so-called NM phase of KMF_3_ (M = Ni, V) where spin-up and spin-down channels show the same electron distribution. As shown in Table 1 (and Figures S2 and S3 in the Supporting Information), this phase is unstable and, upon allowing spin-polarization, the electrons undergo a redistribution and a stabilization energy Δ*E* is obtained when moving toward the insulating FM or AFM phases, as predicted by Hubbard’s and Anderson’s models. However, a detailed examination of the energy contributions (Table 1) and the changes in total density (Figure 1) reveals the different behavior in systems with unpaired σ and π electrons. In KNiF_3_, the reduction of energy going from NM to FM or AFM is dominated by a decrease in electron–electron repulsion (*V*_ee_) (Table 1) and a main transfer of electron density from Ni^2+^(dσ) → F^–^(pσ) (Table 2 and Figure 1) accompanied by a smaller π-backdonation, F^–^(pπ) → Ni^2+^(dπ), the so-called Dewar, Chatt and Duncanson process.^26–28^ Moreover, these changes in the electron density of the insulating phases (see Figure 1) force a simultaneous increase of the kinetic energy *T*(FM/AFM) > *T*(NM) and electron–nuclear potential energy *V*_en_(FM/AFM) > *V*_en_(NM). This is in good agreement with the usual image provided by Hubbard’s or Anderson’s models. Interestingly, when comparing FM and AFM phases of KNiF_3_ (Table 1) the sign of Δ*E* is the same as that of Δ*T*, a trend that is also observed in KVF_3_. We have verified that this conclusion also holds when varying the lattice parameter.

**Figure 1:**
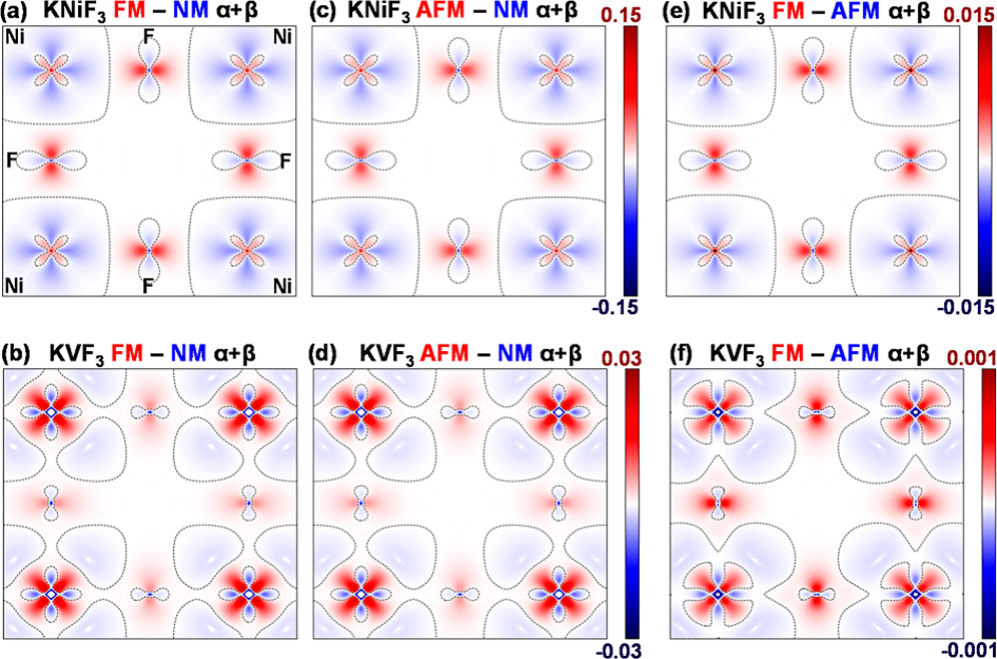
Difference electron densities ρ_D_ FM – NM (a,b), AFM – NM (c,d) and FM – AFM (e,f) obtained for KNiF_3_ (top) and KVF_3_ (bottom) on the (001) plane. Black dashed lines correspond to ρ_D_ = 0. The scale for FM – NM and AFM – NM differences is the same.

Regarding the instability of the NM phase in KVF_3_, it is not driven by a decrease in *V*_ee_, like in those models, but rather due to the insufficient attraction of the electrons by the nuclei in the NM phase (Table 1). The opposite behavior to KNiF_3_ is also observed in the charge transfer, where a V^2+^(dπ) → F^–^(pπ) transfer and a subsequent σ-backdonation F^–^(pσ) → V^2+^(dσ) occurs. As a result (Figure 1) the three t_2g_ electrons become more localized around V^2+^ in the magnetic phases when comparing to the NM one, producing a large increase of *V*_ee_ that is compensated by a reduction of *V*_en_ (i.e., *V*_en_ becomes more negative). It is important to note that models including only the magnetic orbitals cannot account for the backdonation (either in KNiF_3_ or KVF_3_), as their minimal basis is not prepared to describe this phenomenon. In the original L–D paper^11^ on elemental 3d metals, as well as in later work^29^ addressing not only 3d metals but also other systems such as Heusler alloys and quaternary intermetallic borides, it was found that the presence of antibonding states near the Fermi energy in the NM state was the hallmark of the instability of this phase. Here, we find in KMF_3_ (M = Ni, V) that the COHP for the M-F interactions displays this characteristic (see Section S3 in the Supporting Information) although, in addition to results consistent with those of L–D, we find that the COHP for the M–M interactions exhibit nonbonding character, which we interpret as an indicator for instability toward an AFM state.^11^

Examining the electronic density, ρ, in Figure 1, we find that the difference between the FM phase and the AFM phase, ρ­(FM) – ρ­(AFM) (Figure 1e,f), exhibits a spatial distribution pattern similar to that observed in the difference between the NM and magnetic phases (Figure 1a–d). This similarity is also evident in the energy contributions summarized in Table 1. These values clearly show that, in both KNiF_3_ and KVF_3_, changes in potential energy (*V*_ee_ + *V*_en_) favor the FM state while those in kinetic energy (*T*) favor the AFM state. This points toward the importance of electron delocalization in the stabilization of the latter phase. However, it is worth noting that the quantitative differences are much smaller and subtle than those with NM phases, as reflected in the order-of-magnitude scale reduction of Figure 1e,f and FM-AFM energy differences in Table 1.

The results in Figure 1 for ρ_D_ = ρ­(FM) – ρ­(AFM) in KNiF_3_ can be now analyzed using the canonical models on superexchange, focused on linear σ-bonded metal–ligand–metal symmetric dimers (M^*l*^–F–M^*r*^, where l and r mean left and right M ions, respectively) placed along the *z*-axis. We will focus on the H–T–H model,^3^ based on the description of two MOs with antibonding metal–ligand character depicted in Figure 2a, ϕ_g_ and ϕ_u_, that display, respectively, even and odd parity. They can be expressed as follows

1 $$\phi_{g}=\frac{N_{g}}{\sqrt{2}}\left(d^{l}+d^{r}\right)-\lambda_{s}s$$

2 $$\phi_{u}=\frac{N_{u}}{\sqrt{2}}\left(d^{l}-d^{r}\right)+\lambda_{p\sigma }p_{\sigma }$$

Here d^l^ and d^r^ are d­(3z^2^-r^2^)-orbitals of the l and r metal cations directed along the main *z*-axis, while *p*_σ_ and *s* denote, respectively, the 2p_Z_ and 2s orbitals of F. *N*_g_ and *N*_u_ are the normalization constants and λ_s_ and λ_pσ_ are the mixing coefficients of s and p_σ_ orbitals, respectively. As the 2s–2p gap in free F and F^–^ amounts to 24 eV,^30^ it can be expected that λ_pσ_^2^ ≫ λ_s_^2^ (in KVF_3_, λ_s_^2^ = 0 as MOs display π-character) and thus *N*_g_^2^ > *N*_u_^2^. As both ϕ_g_ and ϕ_u_ are antibonding orbitals, the corresponding one-electron energies, ε_g_ and ε_u_, verify ε_g_ < ε_u_. This whole pattern is well reproduced from first-principles calculations for the symmetric M^l^–F–M^r^ dimer, that yield ε_u_ – ε_g_ values of 0.69 eV (KNiF_3_) and 0.30 eV (KVF_3_). In the H–T–H model, the FM state is given by the Slater determinant |ϕ_g↑_ ϕ_u↑_| while the AFM one involves a strong configuration mixing between |ϕ_g↑_ ϕ_g↓_| and |ϕ_u↑_ ϕ_u↓_|, although |ϕ_g↑_ ϕ_g↓_| is dominant.

**Figure 2:**
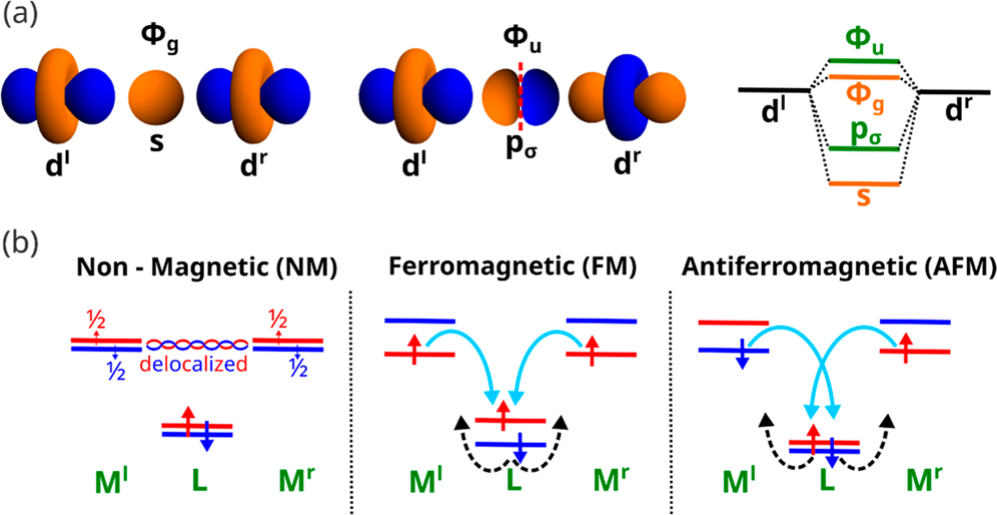
Molecular orbital schemes that describe superexchange. (a) Even and odd molecular orbitals ϕ_g_ and ϕ_u_ and molecular orbital diagram for the symmetric dimer (not to scale). The two lower electronic levels primarily consist of s and p_σ_ ligand orbitals, while the two higher levels correspond to the antibonding MOs ϕ_g_ and ϕ_u_. The main atomic orbitals are d^l^ and d^r^ for the left/right metal d-level, while p_σ_ and s are the ligand σ-orbitals. (b) Qualitative depiction of the α (red) and β (blue) levels for the NM phase and the charge flows produced by the spin polarization in the FM and AFM states. Main metal–ligand donation is described by a light blue arrow and the minority-spin electron delocalization, that is crucial in the stabilization of the magnetic phases, is shown as a dashed black arrow.

Using the H–T–H model, we deduce (see Section S4 in the Supporting Information) that the difference density, ρ_D_, between the triplet (FM phase) and singlet (AFM phase) states is

3 $$\rho_{D}=\rho_{FM}-\rho_{AFM}=\frac{\varepsilon_{u}-\varepsilon_{g}}{U}\left[\phi_{u}^{2}\left(\mathrm{r⃗}\right)-\phi_{g}^{2}\left(\mathrm{r⃗}\right)\right]$$

Here, *U* ≫ ε_u_ – ε_g_ (*U* = 5–10 eV) is the on-site repulsion resulting when an extra electron is placed in an already occupied atomic orbital. According to eqs 1–3, the orbital electron densities close to cation A are given by ϕ_g_^2^ = *N*_g_^2^ *d*_A_^2^/2 and ϕ_u_^2^ = *N*_u_^2^ *d*_A_^2^/2. Therefore, since *N*_g_^2^ > *N*_u_^2^ for the KNiF_3_, ρ_D_ is negative, indicating that the density around the cation is higher in the AFM than in the FM phase, a conclusion in full agreement with our results displayed in Table 2 and Figure 1. Close to the F^–^ ligand, as λ_pσ_^2^ > λ_s_^2^, ρ_FM_ – ρ_AFM_ > 0, so the major contribution corresponds to the FM phase, again in agreement with results in Table 2 and Figure 1. However, around the vertical line passing through the ligand nucleus (see Figure 2a), λ_pσ_ = 0 while λ_s_ ≠ 0, which explains that at the position of the F^–^ ligand ρ_D_ is negative and thus determined by the AFM phase, again as shown in Figure 1.

The results derived from H-T-H model can be also connected with the energy differences shown in Table 1. The wave functions for the triplet ^3^Σ_u_ (FM) and the singlet ^1^Σ_g_ (AFM) state in the H–T–H model are provided in Section S4 of the Supporting Information. In the triplet ^3^Σ_u_ state, the contribution from the odd ϕ_u_ and even ϕ_g_ MOs is the same (50% ϕ_g_ vs 50% ϕ_u_). However, in the singlet ^1^Σ_g_ state, our calculations indicate that the contribution of ϕ_g_ MO is greater than that of the ϕ_u_ MO (60% ϕ_g_ vs 40% ϕ_u_ in KNiF_3_, 55% ϕ_g_ vs 45% ϕ_u_ in KVF_3_). Since the odd ϕ_u_ MO exhibits a more pronounced antibonding character compared to the even ϕ_g_ MO, the value of ∇^2^ϕ is higher in ϕ_u_, leading to a greater kinetic energy *T*(ϕ_u_) > *T*(ϕ_g_). Consequently, the FM state, with a larger contribution from the odd orbital, has a higher kinetic energy compared to the AFM state. Thus, we can see that canonical models offer a nice perspective to understand many of the main trends provided by first-principles simulations. However, there are also some important discrepancies with these models, most notably the unexpected reduction of *V*_en_ in KVF_3_ when allowing for spin-polarization and the significant changes in the density associated to the backdonation.

To gain a deeper understanding of the changes in the full electron density, including contributions from both the antibonding MOs, present in the models, but also deeper bonding orbitals, we have analyzed the electron density differences, ρ_j_(FM) – ρ_j_(NM), ρ_j_(AFM) – ρ_j_(NM) and ρ_D,j_ = ρ_j_(FM) – ρ_j_(AFM), for each spin channel (j = α or β), as shown in Figures 3 and 4 for KNiF_3_ and KVF_3_, respectively. These differences are mapped in the spatial region of a M^l^–F–M^r^ dimer to simplify a detailed inspection of the two M^l^–F and F–M^r^ bond regions. It should be noted that DFT-derived α and β densities are subject to known limitations,^31^ particularly regarding quantitative accuracy. Thus, this analysis is complemented with bonding indices −ICOHP and ICOBI, included at the end of this section. We will focus first on the analysis of the plots for KNiF_3_ (similar conclusions can be extracted for KVF_3_, see Figure 4) and then we will discuss the differences between the two systems.

**Figure 3:**
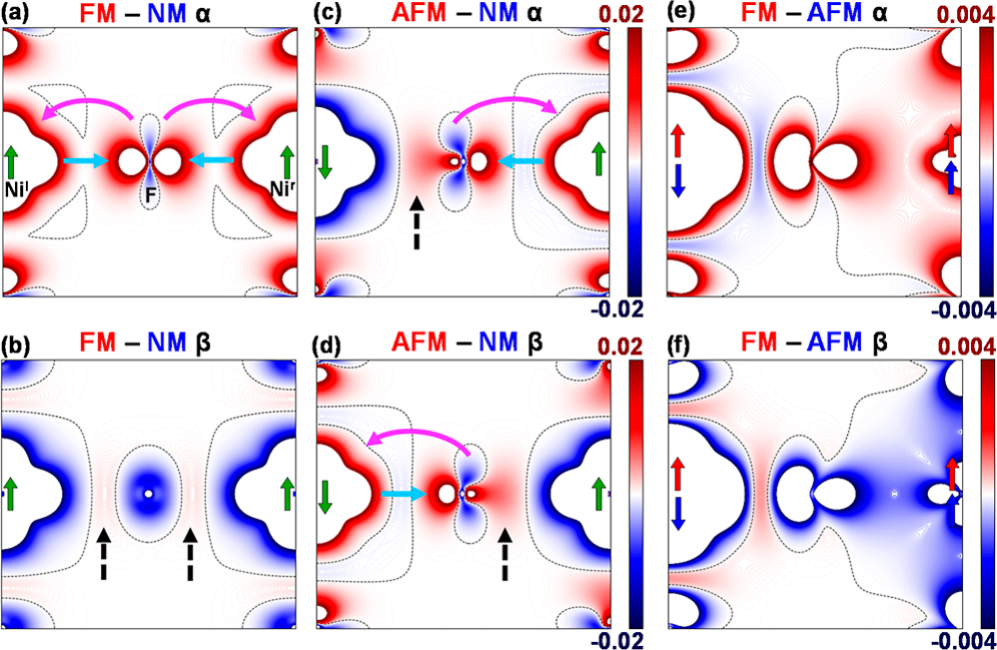
Electron density differences FM – NM (a,b), AFM – NM (c,d) and FM – AFM (e,f) in KNiF_3_ along the Ni^l^–F–Ni^r^ bond line, represented by spin channel. The l and r superscripts denote the left and right Ni cations, respectively. The maps on the first row show the α density, while the β densities are shown in the second row. The black dashed line indicates where the difference is zero. Green arrows at Ni positions indicate the spin directions for the FM and AFM configurations. The FM – NM and AFM – NM maps are represented using the same scale. Light blue arrows indicate charge donation while purple arrows denote charge backdonation occurring in the majority spin regions. Dashed black arrows indicate the charge concentration taking place in magnetic phases in the bond region for minority spin.

**Figure 4:**
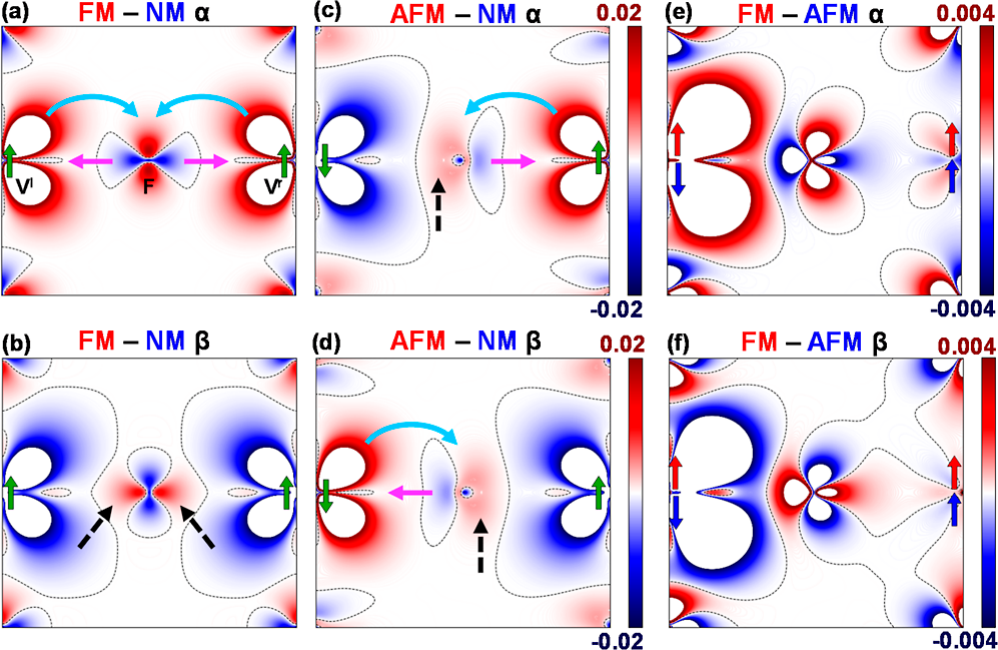
Electron density differences FM – NM (a,b), AFM – NM (c,d) and FM – AFM (e,f) in KVF_3_ along the V^l^–F–V^r^ direction, for α and β spin channels. The maps on the first row show the α density, while the β densities are shown in the second row. The black dashed line indicates where the difference is zero. Arrows at the V positions indicate the spin directions for the FM and AFM configurations. The FM – NM and AFM – NM maps are represented using the same scale. Light blue arrows indicate charge donation while purple arrows denote charge backdonation occurring in the majority spin regions. Dashed black arrows indicate the charge concentration taking place in magnetic phases in the bond region for minority spin.

The main effect of going from the NM to the FM state in KNiF_3_, Figure 3a,b, is the transfer of electrons in the e_g_-band from the β-channel to the α-channel so that the latter has more electrons (spin-α is majority, Figure 3a) and the former fewer than in the NM phase (spin-β is minority) (see Figure 3b). The situation in the AFM state, Figure 3c,d, is similar to the FM one with the exception that the transfer of electrons between channels occurs locally. On the Ni^l^–F bond, α-electrons are transferred to the β-channel while the opposite transfer occurs on the F–Ni^r^ bond. In this way the localization of electrons with complementary spins on opposite sides of the bond in the AFM state involves a partial collapse of the translational symmetry requiring a doubling of the FM-system unit cell. The presence of extra electrons in majority regions for the magnetic phases is clearly visible in Figure 3a,c (right side, F–Ni^r^) and 3d (left side, Ni^l^–F) where the dominance of magnetic phases is represented in red. Similarly, the diminution in the number of electrons in the magnetic phases compared to NM is shown by blue regions in Figure 3b,c (left side, F–Ni^l^) and 3d (right side, Ni^r^–F). KVF_3_ works in a similar way, as shown in Figure 4.

Observing the previous diagrams (Figures 3 and 4) we can see that, in line with many other first-principles calculations^8–11^ but in contrast with other interpretations,^12–14^ that the main effect of the Fermi hole is concentrating the high-density majority spin regions (more parallel spin pairs) in the magnetic phases near the ion nuclei while minority spin regions (less parallel spin pairs) are more diffuse. Careful examination of Figures 3 and 4 reveals two main points that cannot be explained by the classical Anderson/Hubbard models: (i) in the majority spin regions we can clearly observe the backdonation, indicated with purple arrows in Figures 3 and 4, F^–^(pπ) → Ni^2+^(dπ) in KNiF_3_, and, correspondingly, the F^–^(pσ) → V^2+^(dσ) in KVF_3_ (Figures 3a and 4a and the right/left side of Figures 3c and 4c/3d and 4d), while (ii) looking at the minority spin regions we observe that the magnetic phases show larger densities than the NM one in the bond region (indicated with dashed black arrows in the qualitative scheme of Figure 2b and in Figures 3b/4b and the left/right side of Figures 3c and 4c/3d and 4d). This fact is particularly surprising under the light of superexchange models like H–T–H, where there is no contribution to the density from the MOs to the β-channel in the FM phase. Both points (i) and (ii) indicate that there are important changes in the density that are described by orbitals different from the MOs included in the models, in particular, the deep, mostly bonding (ligand) orbitals, whose contributions to the density are important in the metal–ligand bond regions, and the π-t_2g_ orbitals in Ni^2+^ (or σ-orbitals in V^2+^). The former contribution is particularly significant in KVF_3_ (Figure 4) where no occupied d-orbital has a contribution along the V^l^–F–V^r^ bond line (σ direction) but, nevertheless, it shows an important density contribution from the magnetic phases on the minority spin regions. A question that remains unanswered yet is how energetically important are these contributions that are missing in analytical magnetic models. To quantify these contributions, we use the bonding indices -ICOHP^19^ and ICOBI^20^ summarized in Table 3. Both indicate that the one-electron energies are lowest, precisely, on the minority spin regions for both FM (β-channel) and AFM phases (F–M^r^ β-channel and M^l^–F α-channel) in KNiF_3_ or KVF_3_.

**Table 3:** Integrated COBI (ICOBI) and COHP (−ICOHP) Calculated for the Right M^r^–F and Left M^l^–F Bonds of a M^l^–F–M^r^ Dimer in KMF_3_ (M = Ni^2+^, V^2+^)­^a^

	ICOBI				–ICOHP			
state	F–Ni^r^	Ni^l^–F	F–V^r^	V^l^–F	F–Ni^r^	Ni^l^–F	F–V^r^	V^l^–F
NM	0.1802	0.1802	0.2244	0.2244	1.3696	1.3696	1.9466	1.9466
FM α + β	0.1256	0.1256	0.1958	0.1958	1.2646	1.2646	1.8710	1.8710
FM α	0.0481	0.0481	0.093	0.093	0.4926	0.4926	0.8642	0.8636
FM β	0.0775	0.0775	0.1028	0.1028	0.7720	0.7720	1.0071	1.0071
AFM α + β	0.1317	0.1317	0.1972	0.1972	1.2834	1.2834	1.8772	1.8772
AFM α	0.0488	0.0828	0.0940	0.1032	0.5019	0.7816	0.8694	1.0078
AFM β	0.0828	0.0488	0.1032	0.0940	0.7816	0.5019	1.0078	0.8694

a Values for NM, FM and AFM phases are collected, with contributions by spin channel included for the magnetic states.

Let us consider now the FM – AFM α map displayed on Figure 3e. We can see that the FM density dominates around the ions, and the only part of the diagram where AFM is stronger is, precisely, in the bond region dominated by the β spin in the AFM state (Ni^l^–F bond). In KVF_3_ (Figure 4e) we have a similar picture with the addition of a reinforced AFM density along the M^l^–F–M^r^ σ-bond direction, not covered by the idealized models. When observing the β-channel plots (Figures 3f and 4f) there is a complementary pattern of charge concentration around the ions for the AFM state in the regions dominated by the MOs (σ/π for KNiF_3_/KVF_3_, respectively), jointly with an increased density of the FM phase in the bonding region associated with the β-spin Ni^l^–F bond or the M^l^–F–M^r^ σ-direction in KVF_3_. The final result (Figures 1e,f) is the predominance of the AFM phase around the bonding regions associated with the MO but also, and very importantly, around the nodal V^l^–F–V^r^ line of the MOs in KVF_3_. Concerning the bonding indices (Table 3) we observe, again, that the stronger stabilization of the AFM state compared to the FM comes, precisely, from the α-channel (minority) around Ni^l^–F and from the β-channel (minority) in F–Ni^r^. This points to a larger electron-sharing in the AFM than in the FM state. However, the regions where covalency significantly affects the energy are opposite to those expected from the models based only on the MOs.

## Conclusion

In his 1959 work, Anderson already suggested that “there may be a distinct, reasonably universal mechanism for superexchange, and perhaps even that it is related to the Mott mechanism^5^ which prevents conduction”. The results discussed above show that spin polarization, which contains a large part of the electron correlation leading to Mott insulators, produces an electronic instability of the metallic NM phase toward magnetic insulators (Section S3 in the Supporting Information), whose origin can be either an excess of interelectron repulsion (case of KNiF_3_ with σ electrons) or a deficit of electron-nuclei attraction (case of KVF_3_ with π electrons). Although the processes toward FM and AFM states are different, the resulting total electron density is almost the same, with very subtle differences. Spin polarization opens pathways to relax the density, both in the FM and the AFM states, although the latter, localizing electrons with different spin on each side of the metal–ligand–metal bridge, has some further variational freedom,^33^ making it the usual ground state when the M^l^–L–M^r^ angle is equal to 180° and the two M^l^–L and M^r^–L distances are coincident. In cases like K_2_CuF_4_ or Cs_2_AgF_4_, the two distances are different and the ground state is however FM despite displaying an angle of 180°.^34,35^ While the treatment of spin polarization in Kohn–Sham DFT for the AFM phase breaks the symmetry artificially (producing the so-called spin-contamination), we show in the Section S5 in the Supporting Information that the underlying electron localization on opposite sides of the metal–metal bridge is similarly found in multideterminantal methods (like the H–T–H approach). These ideas, led by consecutive symmetry-breaking mechanisms, are well captured by Hubbard’s, Anderson’s or the H–T–H model. Closer inspection of the first-principles density, energy components (*T*, *V*_en_, *V*_ee_) and bond indicators (ICOBI, -ICOHP), however, shows important deviations from these models. In particular, these idealized models do not describe backdonation, even though our simulations indicate that this process is important in the stabilization of the magnetic states. The main shortcoming of these models comes from the use of a minimal basis set that just describes idealized, frozen magnetic orbitals and that (i) does not include the effect of deeper, mostly bonding orbitals that relax in very significant ways, and (ii) does not allow the MOs to change their shape (relax, including more degrees of freedom in their basis as it is usually done in first-principles simulations). Including this electronic relaxation allows to understand why in KVF_3_ the stabilization of the FM or AFM phases from the NM one is steered by the electron–nuclear potential, *V*_en_, which is very rarely (if ever) considered discussing Hubbard’s model, where the electron–electron interaction, in the form of the parameter *U*, is the key to the discussion. In this sense, our work is closely related to the relatively recent revision of the interpretation of Hund’s rule^8–10^ that reached a similar conclusion to the one obtained here. We hope that these findings allow obtaining a clearer understanding of the fundaments behind magnetic interactions in insulators.

## Supplementary Material



## Notes

Computational details for Crystal, VASP and ADF calculations (S1); crystal and magnetic structures and Bader charge analysis (S2); plots of the electronic band structure, density of states and crystal orbital Hamilton populations for the nonmagnetic phases (S3); triplet and singlet densities derived from the Hay, Thibeault and Hoffmann model (S4); and the connection between superexchange models within configuration interaction framework and spin unrestricted DFT (S5) (PDF)
