# Supplementary material for: Magnetic Superexchange and Mott Insulator Mechanisms in Cubic Perovskites: From First-Principles to Canonical Models
Source: Inorg Chem. 2025 Jun 24;64(26):13217–23. doi: 10.1021/acs.inorgchem.5c01522 (PMC12239065; doi:10.1021/acs.inorgchem.5c01522)
Supplement: Supplementary file 1 [file ic5c01522_si_001.pdf]

Supporting Information

Magnetic Superexchange and Mott Insulator Mechanisms in Cubic Perovskites: From First Principles to Canonical Models

Inés Sánchez-Movellán[a], Toraya Fernández-Ruiz[a], Richard Dronskowski[b], Ángel Martín-Pendás[c], Pablo García-Fernández[a], Miguel Moreno[a], José Antonio Aramburu*[a]

[a] Departamento de Ciencias de la Tierra y Física de la Materia Condensada, Universidad de Cantabria, Avenida de los Castros s/n, 39005 Santander, Spain

[b] Institute of Inorganic Chemistry, RWTH Aachen University, Aachen, Germany

[c] Departamento de Química Física y Analítica, Universidad de Oviedo, Calle Julián Clavería 8, Oviedo 33006, Spain.

*email: aramburj@unican.es

S1. Computational details

The optimized structures for $KNiF_3$ and KVF_3, obtained using Crystal[1] and VASP[2,3] codes, are summarized in Table S1. To accommodate the type-G antiferromagnetic (AFM-G) order, the primitive $Pm\overline{3}m$ cell was expanded into a √2×√2×√2 supercell (containing 10 ions), as shown in Fig. S1 (right). The contributions to the total energy, electron density maps, charges, Crystal Orbital Hamilton Population (COHP)[4] and Crystal Orbital Bond Index (COBI)[5] have been calculated at the experimental structure, with all atoms kept fixed to compare the subtle differences due to magnetic ordering.

In the Crystal package the single particle wave functions are expanded as linear combinations of atomic orbitals which are described through Gaussian type functions.[1] All calculations with Crystal were performed using the hybrid functional PW1PW[6] that includes a 20% of exact Hartree-Fock (HF) exchange and the all-electron triple-ζ polarized basis set developed by Peitinger et al.[7] The total energy was converged to 10^{-8} hartree. Integration on the first Brillouin zone was computed using a dense 8×8×8 grid, which gives a spacing between k points in the reciprocal space of 0.196 Å^{-1}. The truncation criteria for bielectronic Coulomb and exchange series (ITOL1-5) were set to 9 (ITOL1-4) and 18 (ITOL5). These integrals are neglected if the overlap between two atomic orbitals is smaller than 10^{-ITOL}. The electron density maps were calculated using Crystal code, as well as Mulliken[8] charges and energy differences. Similar results can be derived from VASP calculations.

In VASP software the Bloch functions expand into plane waves. Calculations with VASP were performed using a 520 eV energy cutoff for valence electrons while the core electrons were included using the projector augmented wave (PAW)[9] method and pseudopotentials. Electron correlation was included through Liechtenstein's[10] DFT+*U* formalism using U = 4 eV. The results derived from PBE+*U* calculations were compared with hybrid functionals PBE0[11] and HSE06[12] (with a 25% of HF exchange). The convergence criterion for electronic optimization was set to 10^{-7} eV. The occupancies for each orbital were obtained by tetrahedron method with Blöchl corrections.[13] The sampling of the reciprocal space was performed using a Γ-centred k-point mesh of 8×8×8.

Despite the usefulness of delocalized plane waves when dealing with periodic solids, these calculations hinder chemical interpretation since, in principle, the local view is lost. However, the locality can be regained as plane waves can be projected into a set of local orbitals. This projection is implemented in LOBSTER[14,15] suite, which starts from a PAW calculation and projects it into a local set of Slater type orbitals (STO). After transforming the delocalized electronic structure into a local orbital basis, the chemical analysis is straightforward. Using LOBSTER, we computed the COHP[4], which decomposes the electronic band structure into orbital-pair interactions, indicating bonding and antibonding contributions to the energy. Integrated COHP (ICOHP) up to the Fermi energy provides an estimation of the bond strength. Recently, the COBI[5] index has been implemented in LOBSTER as an additional bonding descriptor. Unlike the Crystal Orbital Overlap Population (COOP)[16], which relies on electron partitioning, or COHP, based on band structure partitioning, the COBI is directly derived from the density matrix. Integrated COBI (ICOBI) provides a measure of covalent bond order in a solid, eliminating the need for a reference point, in contrast to ICOOP or ICOHP. According to this method, a purely covalent single bond has an ICOBI = 1 while a purely ionic bond corresponds to an ICOBI = 0.

Additionally, calculations on a M–F–M dimer has been performed using the Amsterdam Density Functional (ADF) code[17]. These calculations allowed us to estimate the energy differences between the one-electron levels, mixing coefficients λ_s and $\lambda_{p\sigma}$, and the contributions coming from the even ϕ_g and odd ϕ_u magnetic orbitals (derived using Mulliken population criterion), present in the model by Hay, Thibeault and Hoffmann for superexchange interaction. The ADF program is based on the Kohn-Sham approach to DFT, with basis functions represented by Slater type orbitals (STO). The polyatomic system in ADF is constructed from fragments, i. e., the molecular one-electron orbitals are computed as a linear combination of symmetrized fragment orbitals.

For the dimer calculations, we built a 25 ions cluster ($Ni_2F_{11}K_{12}$) and verified that the embedding, introduced as classical point charges calculated using a mixed Ewald-Evjen[18,19] method, did not produce significant changes in the results. The widely used

B3LYP[20] hybrid functional (20% of exact HF exchange) has been employed in the calculations, combined with triple-ζ polarized basis set. The frozen core approximation was applied for core electrons (1s-3p for Ni^{2+} and for V^{2+} and 1s for F^-).

S2. Geometry, magnetic structure and Bader charges of KMF_3 (M = Ni^{2+}, V^{2+})

Experimental and optimized structures of $KNiF_3$ and KVF_3 are collected in Table S1. Figure S1 shows the AFM-G[21] magnetic ordering in 2×2×2 and √2×√2×√2 supercells.

System	Data	Functional	a (Å)
KNiF3	Exp[22]		4.012
	Crystal	PW1PW	4.019
	VASP	PBE+U	4.053
		HSE06	4.027
KVF3	Exp[23]		4.131
	Crystal	PW1PW	4.135

Table S1. Experimental and calculated lattice parameters for $KNiF_3$ and KVF_3 in the cubic $Pm\overline{3}m$ phase with AFM-G magnetic ordering.

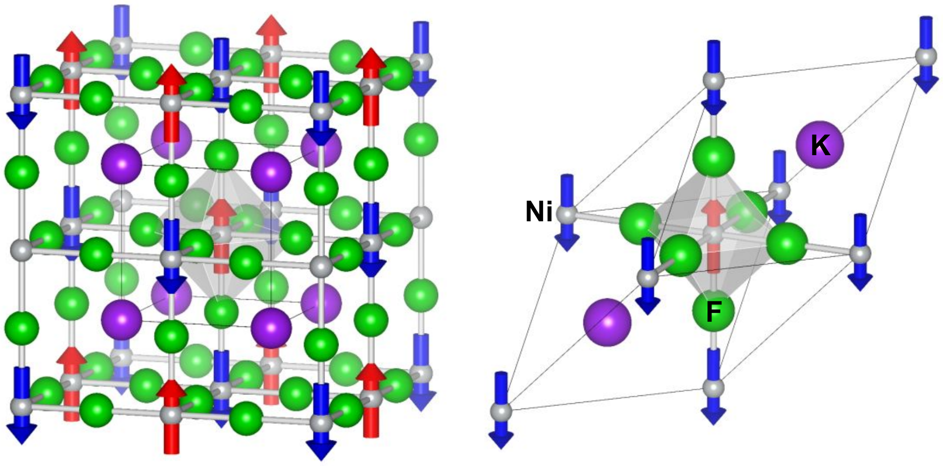

Figure S1. Left: Magnetic structure (AFM-G) of $KNiF_3$ in a 2×2×2 supercell. Right: √2×√2×√2 supercell used in Crystal and VASP calculations.

Since all the calculations were performed at experimental geometry, we ensured that the energy differences between ferromagnetic (FM) and AFM phases, *E*(FM)–*E*(AFM), were consistent among the different functionals. The results are shown in Table S2.

Code	Functional	*E*(FM)-*E*(AFM)
Crystal	PW1PW	0.08
VASP	HSE06	0.07
	PBE0	0.07
	PBE+U	0.09

Table S2. Energy differences (in eV) between FM and AFM states calculated using Crystal and VASP codes with both hybrid and PBE+U exchange-correlation functionals.

For the sake of comparison, the number of electrons on each ion calculated with Löwdin and Bader[24] partitions are collected in Table S3.

		$KNiF_3$				KVF_3			
		**NM**	**FM**	**AFM**	**FM-AFM**	**NM**	**FM**	**AFM**	**FM-AFM**
**Bader**	M^{2+} $N(e^-)$	26.735	26.546	26.560	-0.014	21.468	21.424	21.426	-0.005
	M^{2+} charge	+1.265	+1.454	+1.440	+0.014	+1.532	+1.576	+1.574	+0.005
	F^- $N(e^-)$	9.731	9.793	9.789	+0.004	9.819	9.835	9.834	+0.001
	F^- charge	-0.731	-0.793	-0.789	-0.004	-0.819	-0.835	-0.834	-0.001
**Löwdin**	M^{2+} $N(e^-)$	8.74	8.51	8.53	-0.02	11.66	11.55	11.56	-0.01
	M^{2+} charge	+1.26	+1.49	+1.47	+0.03	+1.34	+1.45	+1.44	+0.01
	F^- $N(e^-)$	7.7	7.77	7.76	+0.01	7.71	7.75	7.75	0.00
	F^- charge	-0.7	-0.77	-0.76	-0.01	-0.71	-0.75	-0.75	0.00

Table S3. Total number of electrons, $N(e^-)$, and ionic charges calculated by Bader criterion with TOPOND[25] for M^{2+} and F^- ions in $KNiF_3$ and KVF_3 for the non-magnetic (NM), FM and AFM phases. Löwdin populations and net charges calculated with LOBSTER for valence orbitals (3d4s in Ni^{2+}, 2s2p in F^- and 3s3p3d4d in V^{2+}) are also gathered.

S3. Band structure, DOS and COHP of KMF_3 (M = Ni^{2+}, V^{2+})

Figs. S2 and S3 represent the band structure calculated with VASP, as well as the density of states (DOS) and COHP computed with LOBSTER for the NM phase of $KNiF_3$ and KVF_3, respectively.

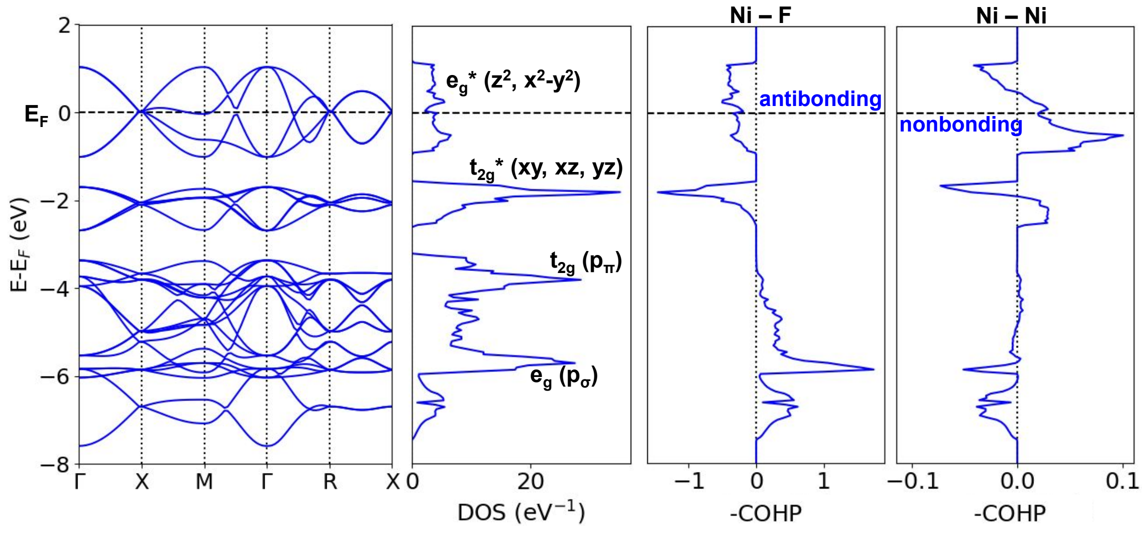

Figure S2. Band structure (VASP), DOS (LOBSTER) and -COHP (LOBSTER) for $KNiF_3$ in the NM phase. The e_g and t_{2g} character of the bands is indicated in the DOS plot. The -COHP is shown for Ni–F and Ni–Ni interaction, with Ni–Ni interaction being one order of magnitude weaker than Ni–F interaction. Energies are shifted to place the Fermi level at zero. These results are consistent with those obtained using Crystal code.

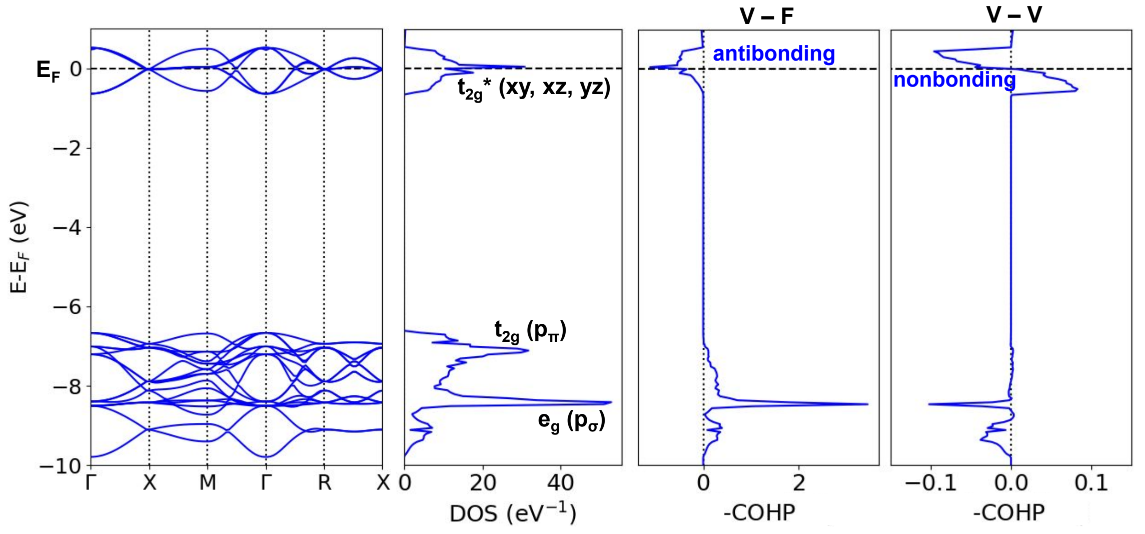

Figure S3. Bands (VASP), DOS (LOBSTER) and -COHP (LOBSTER) for KVF_3 in the NM phase. The e_g and t_{2g} bands are indicated in the DOS. The -COHP is represented for V-F and V-V interaction. The V-V interaction is one order of magnitude smaller than the V-F interaction. The energy is shifted to the Fermi level. Equivalent results can be obtained using Crystal code.

In the NM phase (spin restricted), the 3d e_g orbitals of Ni^{2+} are half occupied, so the Fermi level lies in the e_g band. Under these conditions, the system exhibits metallic character.

In this hypothetical metallic NM phase, the electrons are less localized compared to insulating phases, as indicated by the ICOHPs and ICOBIs values (Table 3). The Ni–F pCOHP in Fig. S2 reveals a strong antibonding character in the e_g bands. The population of these antibonding states at the Fermi level leads to local electronic instabilities. After spin polarization, the spin majority levels stabilize while spin minority levels shift to higher energies, opening a band gap between the valence and conduction bands. This transition from the NM to either FM or AFM phases turns the system into an insulator and is accompanied by an increase of its ionicity as electrons become more localized in magnetic phases (Table 3). This produces a stabilizing effect, lowering the total energy. The preference for an AFM ground state is related to the non-bonding nature of Ni – Ni interaction at the Fermi level.

S4. Comparison with Hay, Thibeault and Hoffmann (H-T-H) model

The starting point for the H-T-H model[26] are two open shell transition metal (TM) cations M^l and M^r (Ni^{2+} ion) separated by a closed shell anion (F^-), which is placed at the same distance from both cations (symmetric dimer). There is one unpaired electron on each TM, placed on d^l and d^r σ-orbitals. The molecular (magnetic) orbitals resulting from the admixture of ligand s and p orbitals with the d orbitals of the cations are ϕ_g and ϕ_u (Eq. 1 and 2, ϕ_1 and ϕ_2 in the original paper), schematically represented in Figure 2.

From ϕ_g and ϕ_u six many-electron configuration arises. H-T-H work with configuration interaction (CI) and therefore the lowest singlet state is obtained from the admixture of the singlet configuration with the two electrons in ϕ_g MO $^1\Sigma_g(g) = \phi_g(\mathbf{r}_1)\ \phi_g(\mathbf{r}_2)$ and the one with the two electrons in ϕ_u MO $^1\Sigma_g(u) = \phi_u(\mathbf{r}_1)\ \phi_u(\mathbf{r}_2)$. In the lowest triplet state $^3\Sigma_u$ (FM) one electron is place in ϕ_g and the other in ϕ_u, and thus the triplet state can be written as $^3\Sigma_u = (1/\sqrt{2})\{\phi_g(\mathbf{r}_1)\ \phi_u(\mathbf{r}_2) - \phi_u(\mathbf{r}_1)\phi_g(\mathbf{r}_2)\}$.

Considering the interaction of the two singlet configurations one can build two different singlet states: the lowest singlet state (ground state GS) $^1\Sigma_g(GS) = (1/\sqrt{2})\{\phi_g(\mathbf{r}_1)\phi_g(\mathbf{r}_2) - \phi_u(\mathbf{r}_1)\phi_u(\mathbf{r}_2)\}$ and the exited singlet state (exited state EE) $^1\Sigma_g(EE) = (1/\sqrt{2})\{\phi_g(\mathbf{r}_1)\phi_g(\mathbf{r}_2) + \phi_u(\mathbf{r}_1)\phi_u(\mathbf{r}_2)\}$.

Once they calculated the triplet and singlet states, H-T-H[26] obtained the energy difference between the triplet and the ground state singlet, which is given by

$$E_S - E_T = -2K_{ab} + (\varepsilon_u - \varepsilon_g)^2/U \qquad \text{(S.1)}$$

Where K_{ab} is the exchange integral in terms of the orthogonal localized molecular orbitals, which are essentially a d orbital of each TM with a small admixture of ligand orbitals and $U >> \varepsilon_u - \varepsilon_g$ is the on-site Coulomb repulsion.

In our work, we are interested in the small differences in the electronic density between the FM and the AFM phases, so, to compare with our first principles calculations, we have derived the difference electronic density between the triplet (FM) and singlet (AFM) states in the framework of the H-T-H model. The electronic density can be expressed as

$$\rho(\mathbf{r}) = \int |\phi(\mathbf{r}_1, \mathbf{r}_2)|^2 \, d\mathbf{r}_1 \, d\mathbf{r}_2 = 2 \int |\phi(\mathbf{r}_1 = \mathbf{r}, \mathbf{r}_2)|^2 \, d\mathbf{r}_2 \qquad \text{(S.2)}$$

For the triplet state $^3\Sigma_u$ the electronic density is given by

$$\rho_T(\mathbf{r}) = \phi_g^2(r)+\phi_u^2(r) \qquad \text{(S.3)}$$

If the electronic density of the singlet ground state $^1\Sigma_g$(GS) is calculated without considering the admixture with the singlet excited state $^1\Sigma_g$(EE), the resulting density is the one obtained for the triplet state (Eq. S.3). However, when the small admixture with the excited state $^1\Sigma_g$(EE) is included, we obtain:

$$\rho_S(\mathbf{r}) = \phi_g^2(r)+\phi_u^2(r) + \frac{2(\varepsilon_u-\varepsilon_g)}{U}[\phi_g^2(r) - \phi_u^2(r)] \qquad \text{(S.4)}$$

Considering Eq. S.3 and S.4, the difference between the electron density in the triplet (FM) and the singlet (AFM) states is the one given by Eq. (3).

S5. Connection between superexchange models (CI) and spin unrestricted DFT (UDFT)

Spin localization can be determined using both superexchange models based on configuration interaction (CI) and spin unrestricted DFT calculations (UR). We will show that the results from both approaches are equivalent.

Starting with CI models[27], we consider two atomic orbitals (AOs), χ_a and χ_b, centered on the two magnetic ions. The interaction between these orbitals result in two molecular orbitals (MOs): ϕ_g = (1/√2)(χ_a+χ_b) and ϕ_u = (1/√2)(χ_a-χ_b). The ground state wavefunction is a linear combination of the corresponding Slater determinants

$$\psi_{CI} = \frac{\cos\theta}{\sqrt{2}}\begin{vmatrix}\phi_g^{\uparrow}(\vec{r}_1) & \phi_g^{\downarrow}(\vec{r}_1)\\ \phi_g^{\uparrow}(\vec{r}_2) & \phi_g^{\downarrow}(\vec{r}_2)\end{vmatrix} + \frac{\sin\theta}{\sqrt{2}}\begin{vmatrix}\phi_u^{\uparrow}(\vec{r}_1) & \phi_u^{\downarrow}(\vec{r}_1)\\ \phi_u^{\uparrow}(\vec{r}_2) & \phi_u^{\downarrow}(\vec{r}_2)\end{vmatrix} \qquad \text{(S.5)}$$

Expanding the determinants, substituting the MOs with their expressions in terms of χ_a and χ_b, and regrouping the Slater determinants, the CI wavefunction can be written as

$$\psi_{CI} = \frac{1}{2}\begin{bmatrix}(\cos\theta + \sin\theta)\left(\begin{vmatrix}\chi_a^{\uparrow}(\vec{r}_1) & \chi_a^{\downarrow}(\vec{r}_1)\\ \chi_a^{\uparrow}(\vec{r}_2) & \chi_a^{\downarrow}(\vec{r}_2)\end{vmatrix} + \begin{vmatrix}\chi_b^{\uparrow}(\vec{r}_1) & \chi_b^{\downarrow}(\vec{r}_1)\\ \chi_b^{\uparrow}(\vec{r}_2) & \chi_b^{\downarrow}(\vec{r}_2)\end{vmatrix}\right) + \\ (\cos\theta - \sin\theta)\left(\begin{vmatrix}\chi_a^{\uparrow}(\vec{r}_1) & \chi_b^{\downarrow}(\vec{r}_1)\\ \chi_a^{\uparrow}(\vec{r}_2) & \chi_b^{\downarrow}(\vec{r}_2)\end{vmatrix} + \begin{vmatrix}\chi_b^{\uparrow}(\vec{r}_1) & \chi_a^{\downarrow}(\vec{r}_1)\\ \chi_b^{\uparrow}(\vec{r}_2) & \chi_a^{\downarrow}(\vec{r}_2)\end{vmatrix}\right)\end{bmatrix} \qquad \text{(S.6)}$$

In Eq. (S.6), the first two determinants represent ionic contributions, with two electrons localized on one atom, while the last two represent covalent contributions, with one electron on each atom.

For the configuration where both electrons occupy the ϕ_g MO, $|\phi_g^{\uparrow}, \phi_g^{\downarrow}|$, θ = 0 or π (Eq. S.5) and the ionic and covalent terms contribute equally. The same applies when both electrons are placed in the ϕ_u orbital, $|\phi_u^{\uparrow}, \phi_u^{\downarrow}|$, with θ = ± π/2 (Eq. S.5). These configurations represent delocalized solutions. A pure AFM solution arises from the mixing of these two configurations and corresponds to the lower covalent terms in Eq. S.6, thus θ = - π/4.

Considering now the unrestricted wavefunction[27], it can be expressed in terms of the symmetry broken MOs ϕ_i and ϕ_j

$$\psi_{UR} = \begin{vmatrix} \phi_i^{\uparrow}(\vec{r}_1) & \phi_j^{\downarrow}(\vec{r}_1) \\ \phi_i^{\uparrow}(\vec{r}_2) & \phi_j^{\downarrow}(\vec{r}_2) \end{vmatrix} \tag{S.7}$$

$$\phi_i = \frac{1}{\sqrt{2}}\left[\cos\left(\varphi + \frac{\pi}{4}\right)\chi_a + \sin\left(\varphi + \frac{\pi}{4}\right)\chi_b\right] \tag{S.8}$$

$$\phi_j = \frac{1}{\sqrt{2}}\left[\sin\left(\varphi + \frac{\pi}{4}\right)\chi_a + \cos\left(\varphi + \frac{\pi}{4}\right)\chi_b\right] \tag{S.9}$$

As for CI wavefunction, the solutions are fully delocalized when φ = 0, ± π/2, π and localized if φ = ± π/4, ± 3π/4.

To quantify spin localization, the square modulus of the spin on one of the localized orbitals can be used. This operator measures how the magnitude of the local spin increases when projected onto one of the local orbitals. It is defined as follows:

$$\hat{S}_a^2 = \hat{\vec{S}}_a \hat{\vec{S}}_a = \hat{s}_1^2 |\chi_a(\vec{r}_1)\rangle\langle\chi_a(\vec{r}_1)| + \hat{s}_2^2 |\chi_a(\vec{r}_2)\rangle\langle\chi_a(\vec{r}_2)| + \hat{s}_1^2 \hat{s}_2^2 |\chi_a(\vec{r}_1)\rangle\langle\chi_a(\vec{r}_1)| \, |\chi_a(\vec{r}_2)\rangle\langle\chi_a(\vec{r}_2)| \tag{S.10}$$

Evaluating this operator (Eq. S.10) for the two wavefunctions ψ_{CI} (S.6) and ψ_{UR} (S.7), assuming that the overlap between local atomic orbitals $\langle \chi_a | \chi_b \rangle$ = 0, it is obtained

$$\left\langle \psi_{CI} \middle| \hat{S}_a^2 \middle| \psi_{CI} \right\rangle = -\frac{3\hbar^2}{4} \sin 2\theta \tag{S.11}$$

$$\left\langle \psi_{UR} \middle| \hat{S}_a^2 \middle| \psi_{UR} \right\rangle = \frac{3\hbar^2}{4} \sin^2 2\varphi \tag{S.12}$$

Eqs. (S.11) and (S.12) demonstrate that both models lead to equivalent results: a local non-zero spin emerges on each atom when the electrons become localized, resulting in an AFM state.

References

(1) Erba, A.; Desmarais, J. K.; Casassa, S.; Civalleri, B.; Donà, L.; Bush, I. J.; Searle, B.; Maschio, L.; Edith-Daga, L.; Cossard, A.; Ribaldone, C.; Ascrizzi, E.; Marana, N. L.; Flament, J.-P.; Kirtman, B. CRYSTAL23: A program for Computational Solid State Physics and Chemistry, *J. Chem. Theory Comput.* **2023**, *19*, 6891–6932.

(2) Kresse, G.; Hafner, J. Ab initio molecular-dynamics simulation of the liquid-metal-amorphous-semiconductor transition in germanium, *Phys. Rev. B* **1994**, *49*, 14251–14269.

(3) Kresse, G.; Furthmüller, J. Efficient iterative schemes for ab initio total-energy calculations using a plane-wave basis set, *Phys. Rev. B* **1996**, *54*, 11169–11186.

(4) Dronskowski, R.; Blöchl, P. E. Crystal Orbital Hamilton Populations (COHP). Energy-Resolved Visualization of Chemical Bonding in Solids Based on Density-Functional Calculations, *J. Phys. Chem.* **1993**, *97*, 8617–8624.

(5) Müller, P. C.; Ertural, C.; Hempelmann, J.; Dronskowski, R. Crystal Orbital Bond Index: Covalent Bond Orders in Solids, *J. Phys. Chem. C* **2021**, *125*, 7959–7970.

(6) Bredow, T.; Gerson, A. R. Effect of exchange and correlation on bulk properties of MgO, NiO and CoO, *Phys. Rev. B* **2000**, *61*, 5194–5201.

(7) Peintinger, M. F.; Oliveira, D. V.; Bredow, T. Consistent Gaussian basis sets of triple-zeta valence with polarization quality for solid-state calculations, *J. Comput. Chem.* **2013**, *34*, 451–459.

(8) Mulliken, R. S. Electronic Population Analysis on LCAO-MO Molecular Wave Functions. I, *J. Chem. Phys.* **1955**, *23* (10), 1833–1840.

(9) Blöchl, P. E. Projector augmented-wave method, *Phys. Rev. B* **1996**, *50*, 17953–17979.

(10) Liechtenstein, A. I.; Anisimov, V. I.; Zaanen, J. Density-functional theory and strong interactions: Orbital ordering in Mott-Hubbard insulators, *Phys. Rev. B* **1995**, *52*, R5467–R5470.

(11) Adamo, C.; Barone, V. Toward reliable density functional methods without adjustable parameters: The PBE0 model, *J. Chem. Phys.* **1999**, *110*, 6158–6170.

(12) Heyd, J.; Scuseria, G. E.; Ernzerhof, M. Hybrid functionals based on a screened Coulomb potential, *J. Chem. Phys.* **2003**, *118*, 8207–8215.

(13) Blöchl, P. E.; Jepsen, O.; Andersen, O. K. Improved tetrahedron method for Brillouin-zone integrations, *Phys. Rev. B* **1994**, *49*, 16223–16232.

(14) Maintz, S.; Deringer, V. L.; Tchougreeff, A. L.; Dronskowski, R. LOBSTER: A tool to extract chemical bonding from plane-wave based DFT, *J. Comput. Chem.* **2016**, *37*, 1030–1035.

(15) Nelson, R.; Ertural, C.; George, J.; Deringer, V. L.; Hautier, G.; Dronskowski, R. LOBSTER: Local orbital projections, atomic charges, and chemical-bonding analysis from projector-augmented-wave-based density-functional theory, *J. Comput. Chem.* **2020**, *41*, 1931–1940.

(16) Hoffmann, R. How chemistry and physics meet in the solid state, *Angew. Chem. Int. Ed.* **1987**, *26* (9), 846–878.

(17) te Velde, G.; Bickelhaupt, F. M.; Baerends, E. J.; Fonseca Guerra, C.; van Gisbergen, S. J. A.; Snijders, J. G.; Ziegler, T. Chemistry with ADF, *J. Comput. Chem.* **2001**, *22*, 931–967.

(18) Van Gool, W.; Piken, A. G. Lattice self-potentials and Madelung constants for some compounds: Part 1, *J. Mater. Sci.* **1969**, *4*, 95–104.

(19) Tosi, M. P. Cohesion of ionic solids in the Born model, *Solid State Phys.* **1964**, *16*, 1–120.

(20) Stephens, P. J.; Devlin, F. J.; Chabalowski, C. F.; Frisch, M. J. Ab Initio Calculation of Vibrational Absorption and Circular Dichroism Spectra Using Density Functional Force Fields, *J. Phys. Chem.* **1994**, *98*, 11623–11627.

(21) Scatturin, V.; Corliss, L.; Elliott, N.; Hastings, J. Magnetic structures of 3d transition metal double fluorides, *Acta Cryst.* **1961**, *14*, 19–26.

(22) Kijima, N.; Tanaka, K.; Marumo, F. Electron-density distributions in crystals of $KMnF_3$ and $KNiF_3$, *Acta Cryst. B* **1983**, *39*, 557–559.

(23) Williamson, R. F.; Boo, W. O. Lower Valence Fluorides of Vanadium. 1. Synthesis and Characterization of $NaVF_3$, KVF_3, and $RbVF_3$, *Inorg. Chem.* **1977**, *16*, 646–648.

(24) Bader, R. *Atoms in Molecules: A Quantum Theory*, Oxford University Press, USA 1994.

(25) Cossard, A.; Desmarais, J. K.; Cassasa, S.; Gatti, C.; Erba, A. Orbital Interactions in Metal Dimer Complexes, *J. Phys. Chem. Lett.* **2021**, *12*, 1862–1868.

(26) Hay, P. J.; Thibeault, J. C.; Hoffmann, R. Orbital Interactions in Metal Dimer Complexes, *J. Am. Chem. Soc.* **1975**, *97* (17), 4884–4898.

(27) Jensen, F. *Introduction to Computational Chemistry*, John Wiley & Sons Ltd, UK 2007.
